# Advancing genomics and integration of multi-omics for precision oncology using quantum machine learning

**DOI:** 10.1038/s41746-026-02982-y

**Published:** 2026-07-11

**Authors:** Ji-Yong Sung, Jae-Ho Cheong

**Affiliations:** 1 Department of Neurosurgery, Seoul National University Bundang Hospital, Seoul National University College of Medicine, Seongnam-si, Republic of Korea; 2 Department of Surgery, Yonsei University College of Medicine, Seoul, Republic of Korea; 3 Department of Quantum Information Science, Graduate School, Yonsei University, Seoul, Republic of Korea

**Keywords:** Cancer, Computational biology and bioinformatics

## Abstract

Cancer multi-omics faces challenges in handling the scale, complexity, and heterogeneity of multi-omics data, limiting progress in variant interpretation, tumor classification, and modeling cancer evolution. Quantum computing offers a new paradigm using superposition, entanglement, and quantum interference to efficiently explore vast solution spaces. This Perspective highlights how quantum algorithms-such as Quantum Support Vector Machines, Quantum Principal Component Analysis, and quantum generative models-could enhance key tasks in precision oncology, including multi-omics integration, spatial transcriptomics, and neoantigen prediction. Current technical barriers, like qubit noise and limited quantum memory, are discussed alongside strategies to connect quantum computing with biomedical research. Interdisciplinary collaboration will be essential to realizing quantum advantage in cancer multi-omics

## Introduction

Cancer multi-omics seeks to unravel the complex genetic and molecular landscapes that underlie tumor initiation, progression, metastasis, and therapeutic resistance. Over the past two decades, landmark projects such as The Cancer Genome Atlas (TCGA) and the International Cancer Genome Consortium (ICGC) have profoundly expanded our understanding of cancer biology. These consortia have generated vast volumes of multi-dimensional omics data-including genomic, transcriptomic, epigenomic, proteomic, and metabolomic profiles-across diverse tumor types and patient cohorts. These datasets have enabled the identification of driver mutations, classification of molecular subtypes, prediction of prognosis, and discovery of therapeutic targets, thereby laying the foundation for precision oncology^1–4^.

However, the integration and interpretation of such high-dimensional and heterogeneous datasets remain a formidable challenge. The rapid increase in data volume, complexity, and noise is intensifying the computational challenges faced by existing analytical workflows. Key problems such as variant prioritization, integrative multi-omics analysis, tumor heterogeneity modeling, and predictive biomarker discovery demand scalable and efficient computational frameworks. Traditional bioinformatics approaches, often rooted in classical statistics and machine learning, are increasingly strained by these demands. Limitations in data dimensionality reduction, combinatorial optimization, and non-linear modeling hinder our ability to derive actionable insights from the available information^5^.

Quantum computing represents a paradigm shift in computation, offering novel ways to process information by leveraging the counterintuitive principles of quantum mechanics-superposition, entanglement, and quantum interference. Unlike classical bits, which exist in binary states (0 or 1), quantum bits (qubits) can exist in linear combinations of states, enabling the simultaneous exploration of a vast number of configurations. This intrinsic parallelism allows quantum algorithms to tackle problems that are computationally intractable on classical hardware, particularly in domains involving high-dimensional search, complex optimization, and pattern recognition-core tasks in cancer multi-omics^6,7^.

Beyond computational speedups, quantum computing holds unique advantages in modeling biological systems that are quantum mechanical at their core. For instance, accurate simulations of electron transfer in redox enzymes, protein folding dynamics, and molecular interactions between DNA, RNA, and proteins require a quantum description of nature. Understanding these fundamental processes is critical for decoding the molecular mechanisms of oncogenesis and therapy resistance. As such, quantum computing not only offers computational efficiency but also opens the door to novel insights into cancer biology at the quantum level^8^.

In this review, we explore the emerging intersection between quantum computing and cancer multi-omics-an area we refer to as Quantum Cancer multi-omics. We begin by outlining the major computational bottlenecks in current cancer multi-omics research and why quantum-enhanced approaches may be particularly well-suited to address them. We then provide a conceptual overview of quantum computing, including key algorithms and hardware platforms, and examine the early applications of quantum computing in biomedical research. Finally, we discuss current technological limitations, ethical considerations, and propose a translational roadmap for the integration of quantum technologies into cancer research, precision medicine, and clinical oncology^9,10^.

**Fig. 1:**
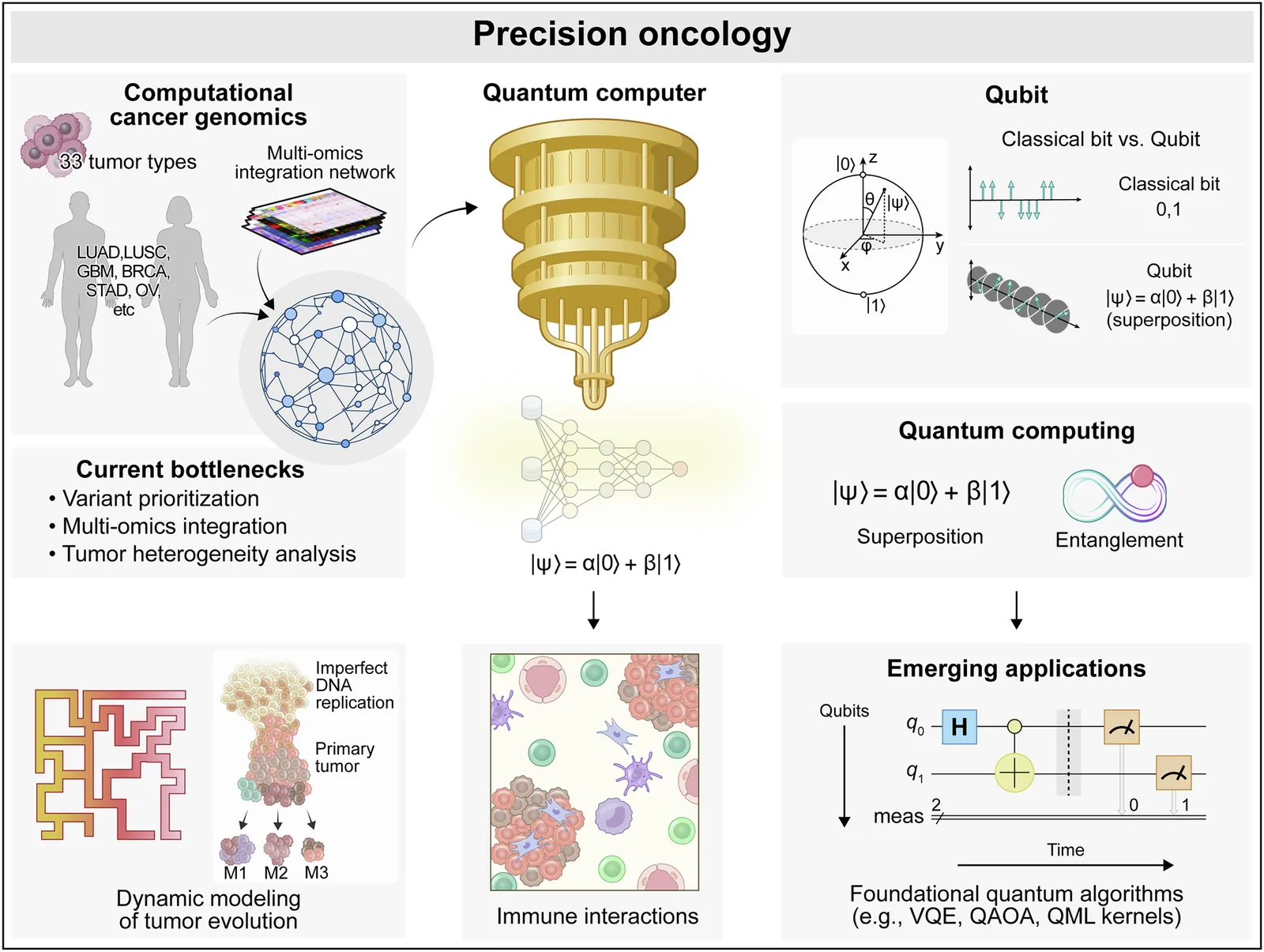
Quantum computing as a transformative tool in computational cancer multi-omics. The integration of quantum computing with computational cancer multi-omicsoffers potential solutions to major bottlenecks in precision oncology, including variant prioritization, multi-omics integration, and tumor heterogeneity analysis. Quantum phenomena such as superposition and entanglement enable novel approaches to dynamic modeling of tumor evolution, immune interactions, and the development of foundational quantum algorithms. These emerging applications aim to accelerate progress in precision oncology through enhanced computational power and new paradigms for understanding cancer biology.

As quantum hardware and algorithms continue to mature, their convergence with omics-driven cancer research may catalyze a new era of biologically informed, computationally empowered precision oncology-bringing us closer to the long-standing goal of truly personalized cancer care^11^ (Fig. 1).

While quantum algorithms are often associated with theoretical computational advantages, such as exponential or quadratic speedups, these benefits are largely established within idealized complexity frameworks. In practice, empirical demonstrations in biomedical contexts, particularly in large-scale cancer multi-omics datasets, remain limited. Therefore, quantum approaches should currently be interpreted as promising but still emerging methodologies rather than mature, clinically deployable solutions.

This review integrates studies published between 2010 and 2025 that explore the intersection of quantum computing and cancer multi-omics. The literature was retrieved from PubMed, Scopus, Web of Science, and arXiv databases using the keywords “quantum computing,” “quantum machine learning,” “bioinformatics,” “multi-omics,” “cancer multi-omics,” and “precision oncology.”

## Challenges in cancer multi-omics demanding quantum solutions

Despite significant advances in sequencing technologies and computational biology, cancer multi-omics continues to face a number of persistent and escalating challenges that strain the limits of classical computation. The intrinsic genomic and phenotypic heterogeneity of cancer, both between patients (inter-tumoral heterogeneity) and within a single tumor (intra-tumoral heterogeneity), underpins much of this complexity. Tumors evolve through dynamic, branching mutational processes that give rise to diverse clonal populations, each with distinct genetic, epigenetic, and transcriptomic profiles. Understanding these landscapes is critical for early detection, prognostication, and therapy selection—but decoding them requires unprecedented levels of computational precision and scalability. One of the foremost obstacles is the discrimination between driver and passenger mutations. Tumor genomes contain thousands of somatic alterations, most of which are neutral or inconsequential. Isolating the relatively few driver events that confer selective advantage requires integrating noisy data across multiple biological levels and patient cohorts. Classical bioinformatics pipelines often employ heuristic filters, statistical thresholds, or machine learning models that struggle with sparse signals embedded in high-dimensional noise^12,13^.

This complexity is compounded when attempting multi-omics integration, which seeks to unify genomic, transcriptomic, epigenomic, proteomic, and metabolomic data to form a coherent, systems-level understanding of tumor biology. These datasets differ in scale, format, resolution, and noise profiles, creating a combinatorial explosion of potential interactions and interpretations. Conventional approaches rely on dimensionality reduction, matrix factorization, or kernel-based methods-techniques that can obscure non-linear relationships and discard biologically meaningful variation.

Recent advances in deep learning, particularly the development of biologically informed neural networks (BINNs)^14^, have significantly improved multi-omics data integration by incorporating prior biological knowledge into neural network architectures.

BINNs and other deep learning models can capture nonlinear dependencies across omics layers and have demonstrated strong performance in dimensionality reduction, feature extraction, and pathway-level interpretation. However, these classical approaches remain constrained by the representational limits of tensor-based feature spaces and the exponential computational cost associated with modeling high-order correlations among heterogeneous molecular datasets.

In addition to biologically informed neural networks (BINNs), a wide range of deep learning architectures have been applied to multi-omics integration, including variational autoencoders for latent-space alignment, graph neural networks that leverage pathway-level biological priors, multimodal convolutional networks, and more recently, transformer-based models for high-dimensional and sparse omics data. These complementary approaches highlight the rapid methodological expansion in multi-omics data fusion beyond BINN-based frameworks.

Quantum machine learning (QML) offers a fundamentally different framework by embedding multi-omics data into high-dimensional quantum Hilbert spaces, where correlations are represented through superposition and entanglement rather than linear tensor products. This enables QML models to efficiently capture nonlinear, entangled, and higher-order dependencies across omics modalities that are often inaccessible to classical networks. Moreover, quantum kernel methods allow the mapping of biological data into exponentially large feature spaces, revealing hidden manifolds and latent relationships between genomic, transcriptomic, and proteomic variables that conventional deep learning may overlook. These properties suggest that QML could complement or even surpass classical deep learning approaches such as BINN when dealing with ultra-high-dimensional, noisy, and hierarchically structured cancer multi-omics datasets^11,15^.

The need for scalable, lossless, and interpretable integration methods is increasingly urgent. Furthermore, predicting treatment response and patient stratification based on complex omics signatures remains an elusive goal. While precision oncology aims to deliver tailored treatments to individual patients at optimal times, existing predictive models frequently struggle to maintain accuracy across varying cancer types and patient populations. This failure is largely due to the inability of classical algorithms to capture non-linear, high-order interactions within and between omics layers-interactions that may underlie mechanisms of drug sensitivity, resistance, and synergy. QML introduces a compelling alternative. By embedding classical data into high-dimensional quantum Hilbert spaces and leveraging quantum entanglement, QML algorithms can capture subtle and complex patterns that evade classical detection. Techniques such as variational quantum classifiers, quantum kernel methods, and quantum Boltzmann machines may offer breakthroughs in biomarker discovery, response prediction, and patient clustering^16^.

Another pressing challenge lies in modeling the temporal and evolutionary dynamics of tumors. Cancer progression is not static but driven by Darwinian selection acting on a heterogeneous population of cells under treatment pressure. Simulating these dynamics-including mutation accumulation, immune evasion, and therapeutic resistance-requires solving high-dimensional stochastic differential equations and Markov processes over vast state spaces. Classical simulation techniques are severely limited by combinatorial scaling. Quantum computing offers a novel solution through quantum simulation frameworks. Quantum walks, quantum annealing, and Hamiltonian-based dynamics can model complex evolutionary trajectories and energy landscapes with exponentially fewer resources. These tools may enable researchers to simulate clonal selection, predict the emergence of resistance-conferring mutations, and design evolution-informed therapeutic regimens^17,18^.

The emergence of spatial transcriptomics and spatial multi-omics technologies introduces a new frontier of data complexity. These platforms provide spatially resolved molecular maps of gene expression, chromatin accessibility, and protein abundance across tissue sections, revealing the architectural organization of the tumor microenvironment (TME). However, integrating spatial data with genomic alterations presents significant analytic hurdles due to the sheer size, multimodal nature, and dimensionality of the data. Quantum-enhanced algorithms, such as quantum tensor decomposition, quantum graph neural networks, and quantum manifold learning, are particularly well-suited to tackle these challenges. These approaches can uncover latent spatial patterns, infer cell-cell communication networks, and resolve molecular heterogeneity at subcellular scales with enhanced computational tractability. Quantum approaches hold promise for capturing the fine-grained structure and behavior of the tumor microenvironment (TME), potentially revealing how spatial organization influences cancer progression and immune system evasion^19–21^.

Unlike other biomedical domains, cancer multi-omics presents unique computational bottlenecks that are inherently well-suited for demonstrating quantum advantage. Tumors evolve as dynamic ecosystems composed of genetically diverse clonal populations that continuously compete, adapt, and respond to therapeutic pressure. Modeling these clonal evolution trajectories requires solving large-scale, stochastic, and high-dimensional systems that quickly exceed the tractability of classical algorithms. Similarly, tumor heterogeneity-both inter-tumoral and intra-tumoral-creates combinatorial complexity across multiple omics layers, making it difficult to capture higher-order dependencies using conventional machine learning approaches.

Moreover, neoantigen landscape prediction and evolutionary therapy resistance modeling involve exploring extremely large molecular configuration spaces, where interactions between mutations, immune recognition, and selective pressures form rugged and non-convex energy landscapes. Quantum algorithms, particularly quantum walks, variational quantum eigensolvers (VQE), and quantum approximate optimization algorithms (QAOA), offer powerful ways to simulate and optimize such landscapes through superposition and entanglement. By enabling the simultaneous exploration of vast mutational and immunological state spaces, quantum computing provides a means to model cancer evolution and immune escape with fidelity and scalability unattainable by classical computation.

Collectively, these cancer-specific challenges-clonal dynamics, heterogeneity, neoantigen complexity, and adaptive resistance-illustrate why cancer multi-omics constitutes a particularly promising testbed for realizing true quantum advantage in biomedical research^22–24^.

In sum, the challenges faced by cancer multi-omicsare not simply issues of data size, but of complexity, structure, and biological nuance. Quantum computing, by offering fundamentally new ways to represent, manipulate, and learn from data, provides a promising frontier for overcoming these obstacles. As the boundaries of classical computation are tested by the demands of multi-omics integration, spatial biology, and tumor dynamics modeling, quantum approaches may hold the key to unlocking a deeper, more predictive understanding of cancer systems biology^25^.

## Foundations of quantum computing for multi-omics

As the computational demands of cancer multi-omics continue to outpace classical capabilities, quantum computing emerges as a revolutionary paradigm that redefines how information is represented, processed, and learned. At its core, quantum computing operates on quantum bits (qubits), which-unlike classical bits-can exist in superpositions of 0 and 1, allowing quantum systems to explore vast solution spaces in parallel. Additionally, qubits can become entangled, enabling non-local correlations and interference patterns that are inaccessible to classical systems. These unique properties enable quantum algorithms to outperform their classical counterparts in specific problem domains, including many that are central to genomics and systems biology.

Several foundational quantum algorithms hold particular relevance for the multifaceted challenges described in cancer multi-omics. Grover’s Search Algorithm. This algorithm provides a quadratic speedup for searching unstructured datasets-such as variant databases or mutation catalogs. In the context of cancer multi-omics, Grover’s algorithm could accelerate the identification of rare driver mutations or specific molecular patterns across high-dimensional genomic datasets, potentially reducing search complexity from O(N) to O(√N)^26^.Quantum Support Vector Machines (QSVMs). By mapping input data into exponentially large quantum feature spaces via kernel methods, QSVMs enable the classification of complex genomic variants, tumor subtypes, or treatment response profiles. The ability to represent and separate non-linearly separable biological data in high-dimensional Hilbert space may dramatically improve classification accuracy in noisy, heterogeneous omics datasets^15^.

Quantum Principal Component Analysis (qPCA). High-throughput genomics routinely generates data with tens of thousands of features. qPCA leverages quantum parallelism to perform dimensionality reduction with high efficiency, preserving critical biological variance. Unlike classical PCA, qPCA can operate in logarithmic time with respect to the size of the data, making it particularly suited for real-time analysis of streaming multi-omics data^27^.

The VQE^28^ and QAOA are promising quantum algorithms for addressing core optimization tasks in systems biology-ranging from reconstructing gene regulatory networks and uncovering cellular community structures to refining patient stratification strategies. While VQE, originally rooted in quantum chemistry, can be repurposed to identify low-energy states in biological models, QAOA offers a versatile toolset for tackling combinatorial challenges in genomics^29^.

These quantum algorithms are typically deployed within hybrid quantum-classical architectures, where quantum processors handle computationally intensive subroutines (e.g., feature mapping, optimization subspaces), while classical hardware manages orchestration, pre-processing, and iterative training. This hybrid model is particularly important in the current Noisy Intermediate-Scale Quantum (NISQ) era, where quantum hardware remains limited by decoherence, gate errors, and qubit connectivity.

Despite current hardware limitations, several proof-of-concept studies have demonstrated the potential of QML across diverse biomedical applications including protein folding prediction via quantum-enhanced optimization, gene expression analysis using quantum classifiers, cancer survival outcome modeling with quantum neural networks, and clustering of single-cell RNA-seq data through variational quantum approaches^30,31^.

One particularly exciting area is the development of quantum generative adversarial networks (QGANs)^32^. These architectures mimic the structure of classical GANs, with a quantum generator and/or discriminator, and have shown promise in synthetically generating rare or missing genomic profiles. This can be especially valuable in cancer multi-omics, where patient heterogeneity and cohort imbalance hinder statistical power. By enriching underrepresented classes in training datasets, QGANs may improve the robustness of AI models used in personalized oncology^21,33^. Moreover, quantum-inspired classical algorithms-such as tensor network methods, quantum annealing heuristics, and simulated quantum walks-are being developed as accessible alternatives that mimic aspects of quantum logic on classical hardware. These algorithms provide an important translational bridge, enabling researchers to begin exploring quantum paradigms in cancer multi-omics today, even in the absence of large-scale fault-tolerant quantum computers^21^.

Taken together, the foundational principles and emerging algorithms of quantum computing offer a radically different computational toolbox-one that is not just faster, but fundamentally better suited to the nonlinear, multiscale, and high-dimensional nature of biological systems. For genomics, this means the possibility of modeling not just static genomic snapshots, but the dynamic, evolving, and spatially complex architectures of cancer, from regulatory networks to spatially resolved tumor ecosystems^11^.

In the next section, we turn to current and emerging applications of quantum computing in cancer multi-omics, highlighting early successes, pilot studies, and the immense untapped potential that lies ahead.

## Applications of quantum computing in cancer multi-omics

With the theoretical foundation established, quantum computing is now beginning to demonstrate tangible impact in core tasks of cancer multi-omics. Its ability to process high-dimensional, nonlinear, and probabilistic biological data opens new frontiers across the entire pipeline-from variant discovery to immunotherapy design. Below, we outline five transformative application domains where quantum approaches may outperform classical methods in both efficiency and biological insight^34,35^.

To avoid overinterpretation of methodological maturity, it is important to distinguish between theoretical proposals, proof-of-concept simulations, and experimentally validated approaches. Throughout this section, we explicitly indicate the level of evidence associated with each application domain. This distinction is particularly important in emerging fields such as quantum biomedicine, where methodological development often precedes large-scale empirical validation.

Table 1 summarizes key application domains where quantum computing (QC) has been or can be applied to cancer multi-omics. Proof-of-concept studies represent demonstrated implementations on small-scale datasets, while future/speculative directions indicate conceptual extensions awaiting experimental validation. Proof-of-concept studies refer to small-scale or simulated demonstrations, while future directions denote conceptual or yet-to-be experimentally validated applications.

**Table 1:** Applications of quantum computing in cancer multi-omics

Application Domain	Quantum Approach / Algorithm	Proof-of-Concept Demonstrations (Existing Studies)	Future / Speculative Directions	Representative References
Variant Prioritization & Classification	Quantum Support Vector Machine (QSVM), Quantum Kernel Methods	QSVM used for breast-cancer subtype classification and mutation feature selection.	Quantum-enhanced ensemble classifiers integrating genomic + clinical data.	^15,88^
Multi-Omics Integration	Quantum Principal Component Analysis (qPCA), Quantum SVD (QSVD)	qPCA applied to small-scale gene-expression datasets for feature reduction.	Real-time multi-omics fusion via hybrid quantum–classical pipelines.	^33,89^
Subtype Discovery & Clustering	Quantum k-Means, Quantum Manifold Learning	Quantum-annealing-based clustering for single-cell RNA-seq data.	Quantum graph neural networks for spatial tumor microenvironment profiling.	^19,34^
Evolutionary Modeling & Therapy Resistance	Variational Quantum Eigensolver (VQE), Quantum Approximate Optimization Algorithm (QAOA), Quantum Walks	Quantum walk models for disease-gene prioritization; VQE-based simulation of biological networks.	Quantum simulation of clonal evolution and adaptive resistance landscapes.	^18,53^
Neoantigen Prediction & Immunotherapy	Quantum Generative Adversarial Network (QGAN), Quantum Docking & Optimization	Early use of quantum-inspired docking for peptide–MHC interaction screening.	Quantum generative models to simulate neoantigen libraries and optimize TCR binding.	^24,32,55^
Spatial Transcriptomics & TME Modeling	Quantum Tensor Decomposition, Quantum Graph Neural Networks (QGNN)	Preliminary QGNN frameworks tested on synthetic spatial data.	Quantum manifold learning to map cell–cell interactions and immune gradients in tumor tissues.	^17,19^

### Variant prioritization and classification

Cancer genomes are riddled with mutations, but only a fraction-driver mutations-confer selective advantage. Identifying these drivers amid a sea of passenger variants remains a core challenge. Quantum search algorithms like Grover’s can accelerate the scanning of large-scale variant databases, reducing computational time for identifying high-priority mutations by quadratic factors. More importantly, QSVMs enable classification of variants in high-dimensional feature spaces derived from sequence conservation, predicted structural effects, and regulatory context. QSVMs can learn complex, nonlinear boundaries that distinguish pathogenic from benign variants with higher accuracy, especially when trained on limited datasets. This makes them ideally suited for rare mutation prioritization in rare cancers or pediatric oncology, where data is sparse but clinical stakes are high^36^.

Current evidence for these approaches is primarily based on proof-of-concept implementations and small-scale studies rather than large-scale clinical validation.

### Multi-omics data compression and integration

Modern cancer research increasingly depends on multi-modal data integration, including genomics, transcriptomics, epigenomics, proteomics, and metabolomics, to create holistic tumor profiles. Beyond BINN, several other deep learning frameworks have been widely adopted for multi-omics integration, such as autoencoder-based dimensionality reduction models, graph neural networks capturing interaction-level dependencies, multimodal fusion architectures, and transformer-based approaches capable of modeling long-range and cross-modal relationships. Incorporating these approaches provides a more complete picture of the computational landscape supporting multi-omics fusion.

However, the curse of dimensionality hampers classical algorithms from efficiently integrating such high-volume datasets without loss of critical variance. Quantum Singular Value Decomposition (QSVD) and qPCA offer exponential advantages in compressing and extracting meaningful features from large, sparse, and noisy data matrices. These techniques preserve biologically relevant variance while reducing computational cost, enabling real-time integration of omics data streams. This facilitates downstream tasks such as clonal evolution reconstruction, longitudinal monitoring of resistance emergence, and spatial transcriptomic alignment across tumor sections^37–40^.

Most quantum-based approaches in this domain have thus far been evaluated in simulated or limited-scale settings, with large-scale validation in real-world multi-omics datasets still lacking.

### Quantum-enhanced clustering and subtype discovery

Accurately distinguishing tumor subtypes is critical to the effective implementation of precision oncology in clinical settings. However, tumors are not monolithic—they comprise dynamic and heterogeneous cell populations, including stem-like^41^, quiescent, and drug-resistant clones^41–43^. Classical clustering methods like k-means often struggle with these non-convex, multi-scale substructures. Quantum-enhanced clustering algorithms, such as quantum k-means (q-means)^44^ and quantum manifold learning, leverage the entangled state space of quantum systems to discover subtle patterns and rare populations. These methods can identify rare cancer cell subtypes, such as mesenchymal-like or stem-like cells^45^ in triple-negative breast cancer, or immunosuppressive CAF subtypes^46,47^

in the tumor microenvironment^48^. Their ability to process latent, non-Euclidean geometries in data enables richer biological classification with implications for prognosis and targeted treatment design^49^.

At present, these methods are largely supported by early-stage or simulation-based studies, and their robustness in clinically relevant datasets remains to be established.

### Simulation of genetic networks and evolutionary landscapes

Understanding tumor progression and treatment resistance requires modeling of gene regulatory networks (GRNs) and evolutionary trajectories under selective pressure. Classical stochastic models, such as Monte Carlo or Markov Chain simulations, become computationally intractable as the network size and mutation space grow^50–52^. Quantum simulation frameworks offer a paradigm shift. By encoding GRNs into Hamiltonians, and simulating their time evolution via VQE or quantum walks, researchers can model how mutations propagate, interact epistatically, and lead to resistance. These simulations can also capture phenotypic plasticity, transcriptional noise, and clonal switching-phenomena critical to understanding adaptive resistance and immune evasion. This enables mechanistic insights into why certain tumors relapse post-therapy and how to design interventions that preemptively block escape pathways^23,53,54^. These applications remain predominantly conceptual or theoretical, with limited empirical validation in biological or clinical contexts to date.

A concise overview of these algorithms, their computational focus, and biomedical relevance is summarized in Table 2.

**Table 2:** Key quantum algorithms for cancer multi-Omics

Algorithm	Computational Focus	Potential Biomedical Applications	Representative References
Grover’s Search Algorithm	Quantum-accelerated database search and pattern recognition	Rapid variant lookup in large genomic databases; identifying mutational signatures	^90^
Quantum Support Vector Machine (QSVM)	Quantum kernel-based classification for high-dimensional data	Cancer subtype classification, variant prioritization, patient stratification	^15,30,88^
Quantum Principal Component Analysis (qPCA)	Quantum eigenvalue estimation for dimensionality reduction	Multi-omics data compression, gene-expression clustering	^27^
Variational Quantum Eigensolver (VQE)	Energy minimization through hybrid variational circuits	Modeling molecular energetics in oncogenic proteins; optimizing drug–target interactions	^18,53^
Quantum Approximate Optimization Algorithm (QAOA)	Quantum optimization of combinatorial problems	Pathway analysis, mutation network optimization, therapy response modeling	^91^
Quantum Generative Adversarial Network (QGAN)	Quantum-enhanced generative modeling	Neoantigen prediction, synthetic biomedical data generation	^24,32,92^
Quantum Walks	Graph traversal and network diffusion using quantum superposition	Disease-gene prioritization, protein–protein interaction mapping	^18^

Table 2 summarizes representative quantum algorithms that are most relevant to cancer multi-omics research. Each algorithm is described by its computational focus, potential biomedical applications, and representative references. The table highlights how quantum approaches-ranging from search and classification (Grover’s search, QSVM) to optimization and generative modeling (QAOA, QGAN)-can address diverse challenges in genomics, including variant discovery, multi-omics integration, network optimization, and drug–target interaction modeling. Collectively, these algorithms form the computational foundation for next-generation quantum pipelines in precision oncology.

### Neoantigen prediction and immunotherapy modeling

Personalized immunotherapy-such as neoantigen vaccines or CAR-T cell design-relies on identifying tumor-specific peptide epitopes that bind to a patient’s MHC molecules and are recognized by T-cells. This requires massive computation across sequence space, binding affinity prediction, and structural modeling. Quantum-assisted pattern recognition techniques have the potential to speed up the discovery of promising neoantigens by integrating analysis of genomic sequences with transcriptomic expression profiles. Quantum generative models can even simulate neoantigen libraries de novo, augmenting training datasets for T-cell receptor (TCR) interaction prediction. Additionally, quantum molecular docking methods may enhance the accuracy of MHC-peptide binding simulations by exploring conformational space more efficiently, improving binding affinity estimation and immunogenicity scoring. In combination, these approaches can enhance personalized immunotherapy pipelines, from initial antigen discovery to downstream TCR-matching and immune repertoire modeling^24,55^. Current developments in this area are largely exploratory, with most approaches demonstrated in preliminary or computational settings rather than experimentally validated pipelines.

## Technological limitations and challenges

While the theoretical and early experimental evidence for quantum computing in cancer multi-omics is compelling, its real-world application remains constrained by significant technological and infrastructural hurdles.

Despite recent progress, the practical deployment of quantum computing in cancer multi-omics still faces several substantial barriers. Hardware limitations remain the most critical challenge, as current NISQ devices possess limited qubit counts, short coherence times, and restricted connectivity, leading to high gate errors and low reproducibility. Even when hybrid quantum–classical models are applied, these physical constraints can severely limit scalability for large omics datasets.

Another major obstacle lies in data encoding and representation. Translating biological information-such as genomic sequences, multi-omics matrices, or molecular networks-into quantum states requires efficient encoding schemes. Current strategies such as amplitude encoding or angle encoding demand exponentially increasing qubit resources, while quantum random-access memory (qRAM) remains largely theoretical due to its hardware complexity and noise sensitivity. Equally important are software integration issues. Existing quantum frameworks (Qiskit, Cirq, Pennylane) are not yet fully compatible with mainstream bioinformatics ecosystems such as Bioconductor, scikit-bio, or TensorFlow-omics. This interoperability gap prevents seamless translation of biological datasets into quantum workflows and hinders cross-platform benchmarking.

To further enhance technical clarity, it is important to acknowledge that the current generation of quantum processors still operates within the NISQ era, where full fault-tolerant quantum computation remains out of reach. Although theoretical frameworks for quantum error correction (QEC) exist, implementing logical qubits requires extensive physical-qubit overhead and remains a major engineering bottleneck. As a result, most biomedical and genomic applications currently depend on error-mitigation techniques rather than full error correction, including methods such as hardware-efficient ansätze, zero-noise extrapolation, and dynamical decoupling, which help reduce decoherence and cumulative gate errors. In parallel, data-encoding bottlenecks continue to represent a central technical constraint. Mapping high-dimensional and heterogeneous multi-omics data into quantum states involves substantial qubit resources, with the cost of amplitude or angle encoding scaling exponentially with dataset size. Promising emerging approaches such as density-matrix mapping and quantum random-access memory (qRAM) may eventually alleviate these issues, but they remain largely conceptual and noise-sensitive at present. Given these realities, only a subset of cancer-genomics tasks can be realistically achieved in the short- to medium-term. Feasible NISQ-era applications include variant prioritization, quantum-kernel-based cancer-subtype classification, and low-dimensional multi-omics feature selection, where datasets are compact enough for near-term hardware. More complex computations-such as clonal-evolution simulation, quantum molecular docking, and multi-omics network optimization-will require the advent of fault-tolerant architectures with thousands of stable qubits. Nevertheless, ongoing progress in hybrid quantum–classical algorithms and quantum-inspired frameworks provides a realistic translational pathway toward incremental quantum advantage in cancer multi-omics^5,55–62^.

As of today, we are still operating within the NISQ era^63^ a phase characterized by quantum hardware that is powerful enough to demonstrate quantum advantage in controlled scenarios but too limited for large-scale deployment in biomedical practice^64^.

Finally, there exists a significant human resource and training gap. The interdisciplinary nature of quantum biomedicine requires expertise across quantum information science, molecular biology, and machine learning-yet few researchers possess deep familiarity with all three domains. Targeted educational programs and collaborative consortia are urgently needed to bridge this divide.

### Hardware constraints: limited qubits, noise, and decoherence

Current quantum processors feature only tens to a few hundred qubits, many of which are not fully error-corrected. Decoherence, the rapid loss of quantum information due to environmental interactions, and gate infidelity-errors during logical operations-significantly limit the depth and fidelity of quantum circuits. These constraints are particularly problematic for cancer multi-omics, where real datasets are high-dimensional, noisy, and require deep quantum circuits for modeling complex biological interactions. Long runtimes and error accumulation render many quantum algorithms impractical at current hardware scales. While progress is being made through error mitigation, hardware-efficient ansätze, and hybrid quantum-classical decomposition, robust, fault-tolerant quantum computers with thousands or millions of logical qubits remain years away^57,58,65^.

### Data encoding and quantum memory bottlenecks

Another fundamental challenge is the quantum data input problem: converting large classical biomedical datasets—like whole-genome sequencing or single-cell multi-omics—into quantum states efficiently. Existing methods for quantum state preparation and quantum RAM (qRAM) are either too slow or physically infeasible at scale, creating a bottleneck that limits practical application. Efficient amplitude encoding or density matrix mapping techniques are needed to ensure that high-dimensional, sparse, and multi-modal biological data can be encoded into quantum systems without incurring exponential overhead. Until these are matured, the performance gains of downstream quantum algorithms may be offset by the prohibitive cost of data loading^66–68^.

### Lack of bioinformatics-ready quantum software and integration

Most existing quantum software frameworks-such as Qiskit (IBM), PennyLane (Xanadu), and Cirq (Google)-have been developed primarily for quantum physics, cryptography, and general-purpose algorithm prototyping. While they support the construction of variational circuits and simulation of quantum dynamics, they lack built-in support for biomedical data structures, such as FASTQ files, BAM/SAM formats, or annotated VCFs, and do not integrate seamlessly with dominant bioinformatics tools like Bioconductor, Scanpy, or Seurat.

This disconnect poses a barrier to adoption by life science researchers and clinicians. To bridge this gap, domain-specific libraries-such as QBio, qOmics^69^, or QuantumPipelines-must be developed to abstract away quantum hardware complexity and provide intuitive interfaces for genomics researchers. This will require sustained cross-disciplinary collaboration between quantum physicists, algorithm designers, and biomedical informaticians^5,25,70^.

### Benchmarking, validation, and reproducibility challenges

One of the most pressing issues is the lack of standardized benchmarking protocols. Without consensus datasets and metrics, it is difficult to rigorously compare quantum algorithms to classical counterparts or to demonstrate quantum advantage in biomedical contexts. To enhance clinical relevance, quantum algorithms should be systematically evaluated using real-world datasets such as The Cancer Genome Atlas (TCGA), large-scale cohort studies, and emerging single-cell multi-omics datasets. Such validation is essential to establish whether quantum approaches provide measurable advantages over classical methods in practical biomedical settings. In contrast, simulations using small synthetic datasets often fail to reflect the true heterogeneity and scale of patient-derived genomic data^71,72^. For example, quantum classification models can be benchmarked against classical machine learning approaches using identical TCGA-derived datasets, with evaluation metrics including predictive accuracy, robustness across cohorts, and computational efficiency.

A critical step toward establishing the credibility and translational value of quantum computing in cancer multi-omics is the systematic benchmarking of quantum algorithms against classical baselines. To move beyond theoretical novelty, performance must be evaluated using standardized metrics and publicly accessible datasets. Representative biomedical resources such as The Cancer Genome Atlas (TCGA), the Pan-Cancer Analysis of Whole Genomes (PCAWG), and emerging single-cell omics consortia provide ideal platforms for open and reproducible benchmarking.

Benchmarking should incorporate quantitative metrics-including predictive accuracy, computational efficiency, model interpretability, and energy cost-to assess whether quantum methods deliver tangible advantages over classical or hybrid approaches. In addition, community-driven benchmarking initiatives, analogous to the DREAM and CASP challenges, are needed to coordinate shared datasets, evaluation standards, and transparent reporting protocols. Such collaborative frameworks would enable fair, reproducible, and regulatorily compliant comparisons between quantum, classical, and hybrid pipelines, thereby clarifying the sui generis contribution of quantum information technologies in precision oncology. To operationalize these goals, the field should develop quantum benchmarking suites tailored to biomedical applications, incorporating open-access, multi-omics reference datasets (e.g., TCGA, PCAWG, and single-cell consortia). Standardized metrics for evaluating predictive accuracy, computational efficiency, and biological interpretability. Shared simulation environments that allow fair, head-to-head comparisons with classical methods.

The creation of such community-wide benchmarking suites would catalyze innovation, enhance reproducibility, and accelerate the identification of quantum algorithms with real translational potential in cancer multi-omics^73,74^.

### Human capital and training gaps

Finally, the successful deployment of quantum computing in cancer multi-omics depends not only on technology but also on people. There is a growing but still limited pool of researchers fluent in both quantum information science and biomedical data science. Without deliberate efforts to train hybrid experts and support collaborative ecosystems, progress will be fragmented. Academic programs, interdisciplinary hackathons, and joint research centers are needed to nurture a new generation of quantum biologists-scientists who understand not only the Schrödinger equation but also the complexities of cancer evolution, immune escape, and systems biology. These individuals will be key to translating theoretical algorithms into usable biomedical tools^11,20,75^.

### Potential solutions and emerging trends

To address these limitations, several technological and methodological trends are emerging. Hybrid quantum–classical workflows are gaining traction, allowing heavy linear-algebra components to run on GPUs or TPUs while delegating complex optimization or sampling tasks to quantum processors. Quantum-inspired algorithms, which mimic quantum behaviors (e.g., tensor contraction, variational optimization) on classical hardware, offer intermediate scalability while preparing data pipelines for future quantum acceleration.

Additionally, new open benchmarking initiatives such as qOmics and QuantumPipelines are being established to provide standardized datasets, reproducible benchmarks, and performance metrics for quantum bioinformatics. These community-driven efforts are essential for comparing quantum algorithms fairly and for promoting transparency and reproducibility in quantum-enabled cancer multi-omics research.

Together, these strategies provide a pragmatic path forward: combining incremental quantum advantage with practical engineering, fostering an ecosystem where hybrid computation, standardized benchmarking, and interdisciplinary training accelerate the transition from theoretical promise to real biomedical impact.

**Fig. 2:**
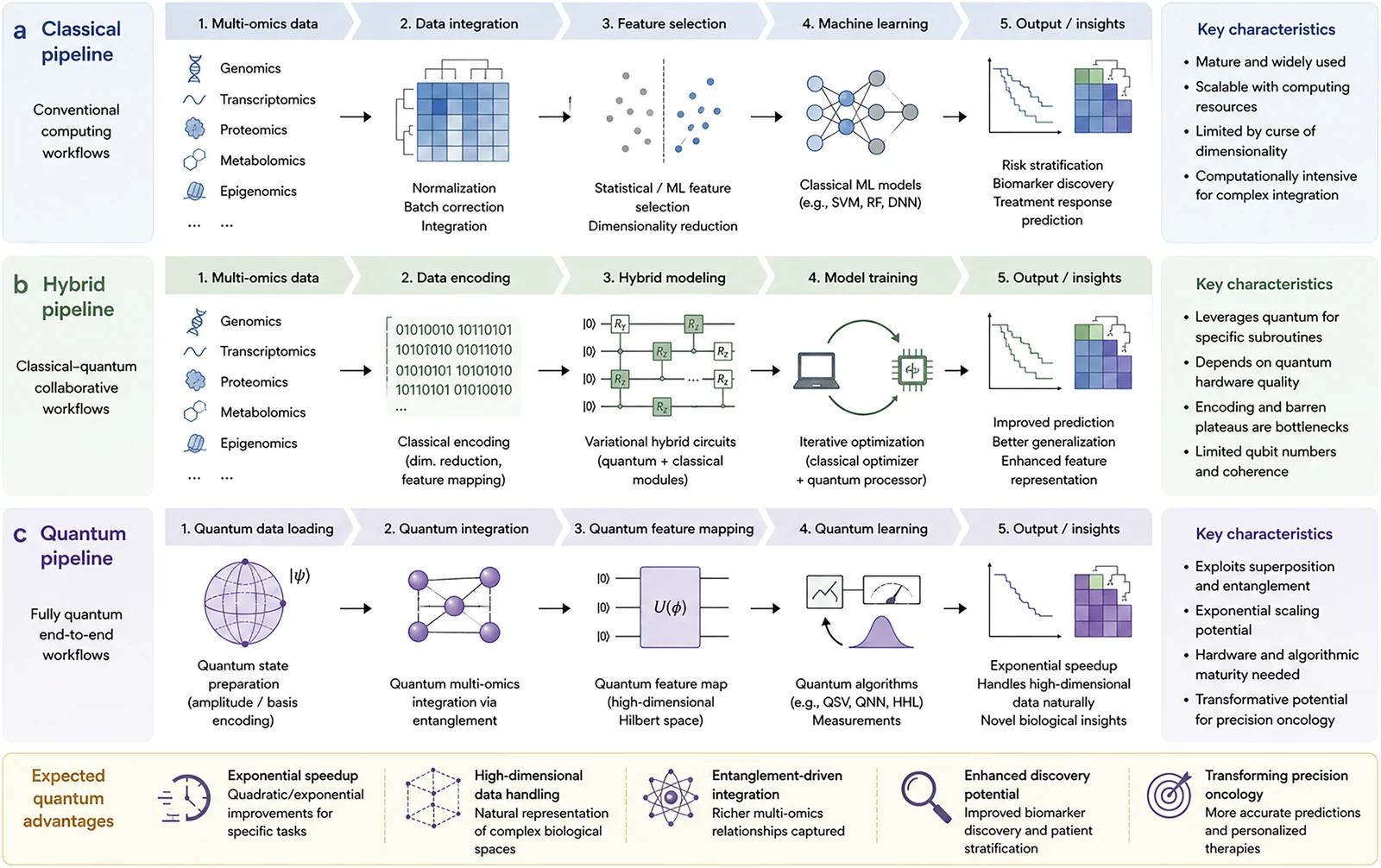
Comparative workflows of classical, hybrid, and quantum pipelines for cancer multi-omics integration. **a** Classical pipeline for cancer multi-omics analysis. Multi-omics data are integrated using conventional computational workflows, followed by feature selection and classical machine-learning models for biomarker discovery, risk stratification, and treatment-response prediction. These approaches are often constrained by computational complexity and the curse of dimensionality. **b** Hybrid quantum -classical pipeline. Classical preprocessing is combined with variational hybrid quantum circuits, where quantum feature mapping and classical optimization are performed iteratively. This framework enhances feature representation while remaining limited by quantum data encoding and current hardware constraints. **c** Fully quantum pipeline. Multi-omics data are encoded into quantum states and processed through quantum integration, quantum feature mapping, and quantum algorithms. By exploiting superposition and entanglement, the workflow enables exploration of exponentially larger search spaces and improved representation of high-dimensional biological data, with potential applications in biomarker discovery, precision oncology, and clinical decision support.

To illustrate the computational transition from conventional to quantum-enhanced paradigms, Fig. 2 presents a comparative workflow encompassing classical, hybrid, and quantum pipelines in cancer multi-omics. The classical pipeline relies on standard data processing, multi-omics integration, and machine learning, which often suffer from computational inefficiency and difficulties in managing high-dimensional data. Hybrid frameworks introduce variational hybrid circuits, combining classical optimization with quantum subroutines to improve feature representation, though they remain constrained by current quantum hardware and encoding complexities. In contrast, the quantum pipeline demonstrates how quantum data loading, feature mapping, and quantum machine learning can potentially deliver exponential speedup and enhanced scalability for multi-omics integration and predictive oncology tasks.

## Future perspectives and clinical translation pathways

As quantum computing technology matures, its integration into cancer multi-omics pipelines has the potential to revolutionize how we understand, diagnose, and treat cancer. The trajectory from theoretical capability to clinical utility, however, requires not only continued hardware and software innovation but also thoughtful co-design with biomedical and clinical stakeholders. Below, we outline future directions and translational strategies necessary to realize the promise of quantum-enabled precision oncology^76^. Importantly, the transition from conceptual and simulation-based studies to data-driven validation using clinically relevant datasets will be a critical step in establishing the real-world utility of quantum approaches in precision oncology.

### Scalable quantum workflows for precision oncology

The next decade will likely see the development of hybrid quantum-classical pipelines that integrate quantum subroutines for specific bottlenecks-e.g., variant prioritization, subtyping, neoantigen prediction-within otherwise classical biomedical workflows. In the near term (3 to 5 years), quantum computing applications in cancer multi-omics are expected to focus on tasks that are compatible with current NISQ era hardware constraints. These include quantum kernel based classification for cancer subtype prediction, low dimensional feature selection and data compression using hybrid quantum and classical workflows, and small scale optimization problems such as variant prioritization or biomarker discovery. Such applications typically involve limited input dimensionality and shallow quantum circuits, making them feasible for implementation on existing quantum devices. In contrast, more complex tasks such as large scale multi-omics integration, evolutionary tumor modeling, and quantum enhanced molecular simulation will likely require fault tolerant quantum systems and thus remain longer term objectives. This staged roadmap highlights a realistic pathway from proof of concept studies to clinically relevant applications. These modular approaches will be critical in the NISQ era, enabling incremental quantum advantage without requiring end-to-end quantum execution. Such pipelines could operate as cloud-based platforms (e.g., via IBM Quantum, Amazon Braket, or Xanadu’s Quantum Cloud), allowing researchers and clinicians to offload quantum tasks like qSVM-based classification or quantum-enhanced clustering without deep expertise in quantum mechanics. Long term, integration into existing genomic analysis ecosystems (e.g., cBioPortal, GDC, or Galaxy) will be essential for mainstream adoption^77,78^.

### Toward clinically relevant quantum biomarkers

One of the most promising translational applications is the discovery of quantum informed biomarkers, molecular signatures whose identification requires quantum processing due to their combinatorial or nonlinear nature. These could include non-classically separable mutational patterns in intratumoral clones, quantum entangled gene expression manifolds representing high-fidelity subtypes, subtle neoantigen-MHC binding conformations best resolved via quantum docking

The ability to capture such biomarkers could enable more accurate patient stratification, real-time treatment adaptation, and prediction of therapeutic resistance-hallmarks of next-generation precision oncology^79^. In a practical validation scenario, quantum-derived biomarkers could be evaluated by comparing their predictive performance against established clinical signatures across independent patient cohorts and survival endpoints.

### Integration with clinical decision support systems (CDSS)

To have real-world impact, quantum-derived insights must be rendered interpretable and actionable within clinical decision-making contexts. This will require integration with Clinical Decision Support Systems (CDSS), electronic health records (EHRs), and tumor boards.

In practical settings, quantum-enhanced workflows are expected to be integrated with existing digital medicine infrastructures, including platforms such as cBioPortal, the Genomic Data Commons, and clinical decision support systems, enabling seamless incorporation of quantum-derived insights into real-world oncology pipelines.

Key enablers include explainable quantum AI (XQAI) techniques that interpret quantum models by tracing their predictions to specific genomic or proteomic features, along with confidence metrics and uncertainty quantification methods derived from variational circuits or quantum Bayesian frameworks, and federated quantum learning architectures that allow hospitals to share quantum models without centralizing sensitive genomic data.

Such systems could recommend personalized therapies based on real-time quantum analyses of a patient’s evolving tumor profile, updated dynamically through longitudinal omics monitoring^8^. For instance, quantum-enhanced predictive models could be incorporated into clinical decision support systems to assist in treatment selection by integrating multi-omics features and generating patient-specific risk or response scores.

### Regulatory, ethical, and data governance considerations

The application of quantum computing in healthcare introduces a new set of ethical, regulatory, and data governance challenges that extend beyond those encountered in conventional biomedical AI. Key questions include how to validate quantum-derived biomarkers or treatment predictions within clinical trials, what standards should govern their integration into clinical decision-making, and how to ensure data privacy, interoperability, and security in distributed or cloud-based quantum computing environments. In addition, addressing potential algorithmic bias and fairness issues in QML models trained on heterogeneous genomic cohorts remains critical.

Addressing these challenges will be essential to ensure the transparency, accountability, and ethical deployment of quantum technologies in precision medicine. Regulatory bodies such as the FDA and EMA should begin developing quantum-specific guidelines in collaboration with industry, academia, and bioethics consortia. Furthermore, standards for model interpretability, reproducibility, and risk assessment must be systematically incorporated into translational pipelines.

To address these questions, quantum workflows must be aligned with established biomedical AI governance frameworks, including FDA Software as a Medical Device (SaMD) guidelines, the General Data Protection Regulation (GDPR) for privacy compliance, and the Global Alliance for Genomics and Health (GA4GH) standards for interoperable data sharing. Embedding QIT pipelines within these regulatory structures will be critical to maintaining accountability and trust across institutions. In addition, ensuring interpretability in quantum models is emerging as a central priority. Approaches such as Explainable Quantum AI (XQAI) aim to map quantum-state representations or circuit parameters to biologically meaningful genomic or molecular features, improving transparency for clinical validation and physician adoption^80–83^.

Finally, the integration of quantum pipelines into Clinical Decision Support Systems (CDSS) will require dedicated risk-assessment and validation procedures, including performance benchmarking, failure-mode analysis, and post-deployment monitoring. These mechanisms parallel established biomedical AI safety protocols and will help ensure that quantum algorithms meet the same standards of efficacy, reliability, and patient safety demanded in precision oncology. Collectively, these considerations underscore that achieving regulatory realism, not only technological innovation, will determine the pace and success of quantum computing’s translation into clinical medicine.

### Collaborative infrastructure and quantum-ready biobanks

Widespread adoption will also require the development of quantum-annotated biobanks and collaborative infrastructures. These may include curated multi-omics repositories designed for quantum benchmarking, containing paired classical and quantum-derived feature sets. International consortia-akin to ICGC or GA4GH-focused on building open-source QML models and validating them across diverse populations. Quantum translational centers embedded within cancer institutes to support clinical trials, hardware access, and clinician education. Such initiatives will accelerate the transition from laboratory research to patient care, ensuring that quantum gains are equitably and efficiently delivered to the clinic.

In summary, quantum computing has the potential to transform cancer multi-omics by solving computationally intractable problems in variant interpretation, tumor evolution modeling, and treatment response prediction. While the path to clinical translation is complex, strategic integration of hybrid workflows, development of interpretable biomarkers, regulatory engagement, and quantum-literate infrastructures can unlock a new paradigm in precision oncology. The fusion of quantum information science with biomedical research is not just a technological evolution-it represents a conceptual leap in how we model, understand, and treat the most complex diseases of our time^6,84^.

To facilitate clinical translation, recent advances are beginning to outline practical pathways that connect quantum computing innovations with real-world oncology workflows. These include quantum-assisted biomarker discovery, where quantum algorithms accelerate feature selection and molecular signature identification; integration with existing biomedical infrastructures such as cBioPortal and the Genomic Data Commons (GDC) to enable hybrid quantum–classical analytics; and the development of quantum-ready biobanks under GA4GH frameworks, ensuring standardized data accessibility and interoperability across institutions.

Collectively, these initiatives form the foundation of a translational roadmap that spans the entire spectrum, from quantum algorithm development and validation to clinical decision support and regulatory approval.

**Fig. 3:**
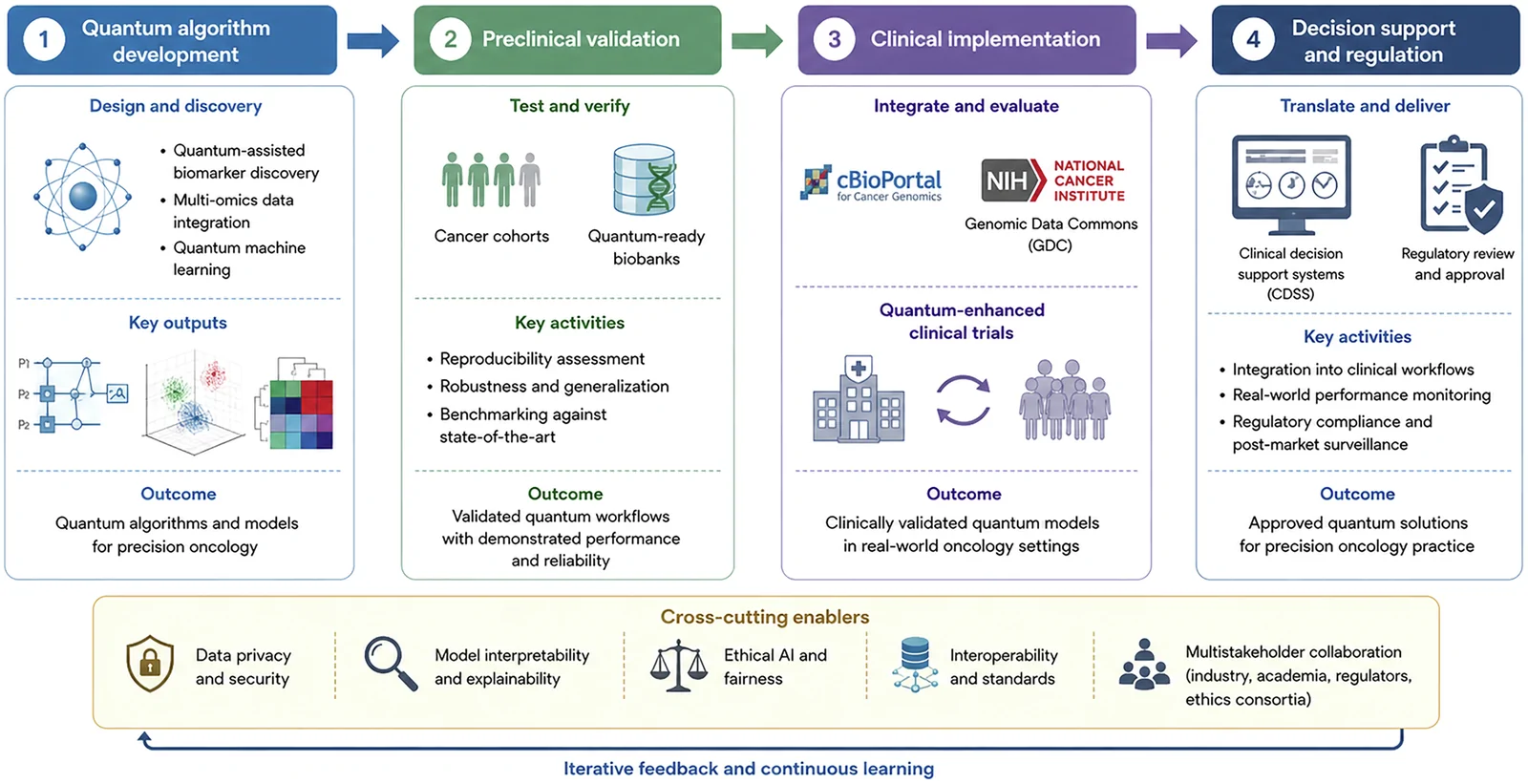
Translational roadmap for quantumenabled cancer multi-omics. The figure illustrates the stepwise pathway for translating quantum computing technologies into clinical oncology applications. The process begins with quantum algorithm development, focusing on quantum-assisted biomarker discovery, multi-omics data integration, and quantum machine learning approaches. Next, preclinical validation involves testing quantum workflows on cancer cohorts and quantum-ready biobanks to ensure reproducibility and robustness. In the clinical implementation stage, quantum models are integrated with biomedical data platforms such as cBioPortal and the Genomic Data Commons (GDC), and evaluated through quantum-enhanced clinical trials. Finally, the decision support and regulation phase includes the incorporation of validated quantum algorithms into clinical decision support systems (CDSS) and formal regulatory approval. Together, these stages form a translational bridge connecting quantum algorithm innovation to real-world precision oncology practice.

This progression is visually summarized in Fig. 3, which illustrates the stepwise integration of quantum technologies into the precision oncology ecosystem. To further ensure scientific reliability and accelerate clinical translation, the development of validation pipelines for quantum algorithms is essential. Because current quantum hardware and algorithms remain in the NISQ stage, validation must proceed through hybrid quantum–classical simulations that combine quantum subroutines with established classical bioinformatics workflows.

In this near-term framework, quantum modules-such as feature mapping, quantum kernel computation, or variational circuit-based optimization-can be integrated into conventional analysis environments (e.g., Seurat or Scanpy for single-cell omics, or cBioPortal for clinical cohort validation). Results generated by quantum models should undergo cross-validation against classical baselines using identical datasets and standardized performance metrics, including predictive accuracy, interpretability, and stability across random seeds and hardware backends. At a later stage, these hybrid validation approaches can evolve into experimental and translational verification pipelines, where quantum-derived predictions (e.g., biomarker patterns, mutational signatures, or drug–target affinities) are tested in in vitro models or compared with clinical-trial outcomes. Such a multilevel validation architecture-from computational cross-checks to experimental confirmation-establishes a realistic pathway toward trustworthy quantum biomedicine, ensuring that quantum discoveries can be replicated, audited, and ultimately certified for clinical use.

## Conclusion

Quantum computing holds the potential to fundamentally reshape the landscape of computational cancer multi-omics. From identifying elusive driver mutations hidden in the noise of vast sequencing data, to modeling the intricate dance of clonal evolution, immune evasion, and therapeutic resistance, quantum algorithms offer a transformative leap beyond the limitations of classical computing. By natively exploiting high-dimensional entanglement, superposition, and non-classical correlations, quantum techniques promise to unlock insights previously inaccessible, offering new strategies for precision diagnosis, prognostication, and therapeutic targeting.

The integration of quantum machine learning into genomics pipelines introduces new capabilities for uncovering subtle, non-linear interactions across genomic, transcriptomic, epigenomic, proteomic, and spatial data modalities. These capabilities are particularly vital in cancer, a disease marked by immense heterogeneity, evolutionary complexity, and high-dimensional data. Through hybrid quantum-classical workflows, even in the NISQ era, researchers can begin leveraging quantum subroutines to accelerate variant prioritization, neoantigen discovery, and subtype classification with enhanced speed and fidelity.

Yet, realizing the full potential of quantum-enhanced cancer multi-omics demands more than technological breakthroughs. A comprehensive, ecosystem-based strategy is needed, encompassing the following elements sustained public and private investment in quantum-biomedical R&D, with dedicated programs supporting use-case development, algorithmic innovation, and hardware access. Development of quantum-ready biomedical infrastructure, including annotated biobanks, benchmark datasets, federated learning architectures, and robust quantum cloud services tailored for life sciences. Cross-disciplinary education and workforce development, equipping a new generation of quantum-literate biologists, clinicians, and data scientists who can navigate the quantum-biology interface with fluency and creativity. Ethical and regulatory frameworks to guide the responsible use of quantum models in medicine, ensuring transparency, fairness, interpretability, and patient trust. Moreover, global collaboration will be crucial to ensure that quantum advances in oncology are inclusive and equitable, transcending geographic, socioeconomic, and institutional boundaries. The formation of international consortia for quantum genomic science, akin to the Human Genome Project, could catalyze shared standards, tools, and discoveries that benefit all.

In conclusion, bridging quantum information science with cancer biology does not merely promise faster computation-it heralds a paradigm shift in how we perceive, interrogate, and ultimately treat one of humanity’s most complex and devastating diseases. The fusion of quantum mechanics with biomedicine opens a new frontier for scientific discovery and clinical translation. With deliberate effort, open collaboration, and visionary leadership, quantum computing can become a foundational pillar of 21st-century precision oncology-transforming not only cancer care but the very nature of biomedical understanding itself^85–87^.

## Data Availability

No datasets were generated or analysed during the current study.
